# Molecular cartooning with knowledge graphs

**DOI:** 10.3389/fbinf.2022.1054578

**Published:** 2022-12-08

**Authors:** Brook E. Santangelo, Lucas A. Gillenwater, Nourah M. Salem, Lawrence E. Hunter

**Affiliations:** Department of Biomedical Informatics, University of Colorado School of Medicine, Aurora, CO, United States

**Keywords:** knowledge graphs, visualization, molecular pathway, graph algorithms, user-centered computing, scientific communication

## Abstract

Molecular “cartoons,” such as pathway diagrams, provide a visual summary of biomedical research results and hypotheses. Their ubiquitous appearance within the literature indicates their universal application in mechanistic communication. A recent survey of pathway diagrams identified 64,643 pathway figures published between 1995 and 2019 with 1,112,551 mentions of 13,464 unique human genes participating in a wide variety of biological processes. Researchers generally create these diagrams using generic diagram editing software that does not itself embody any biomedical knowledge. Biomedical knowledge graphs (KGs) integrate and represent knowledge in a semantically consistent way, systematically capturing biomedical knowledge similar to that in molecular cartoons. KGs have the potential to provide context and precise details useful in drawing such figures. However, KGs cannot generally be translated directly into figures. They include substantial material irrelevant to the scientific point of a given figure and are often more detailed than is appropriate. How could KGs be used to facilitate the creation of molecular diagrams? Here we present a new approach towards cartoon image creation that utilizes the semantic structure of knowledge graphs to aid the production of molecular diagrams. We introduce a set of “semantic graphical actions” that select and transform the relational information between heterogeneous entities (e.g., genes, proteins, pathways, diseases) in a KG to produce diagram schematics that meet the scientific communication needs of the user. These semantic actions search, select, filter, transform, group, arrange, connect and extract relevant subgraphs from KGs based on meaning in biological terms, e.g., a protein upstream of a target in a pathway. To demonstrate the utility of this approach, we show how semantic graphical actions on KGs could have been used to produce three existing pathway diagrams in diverse biomedical domains: Down Syndrome, COVID-19, and neuroinflammation. Our focus is on recapitulating the semantic content of the figures, not the layout, glyphs, or other aesthetic aspects. Our results suggest that the use of KGs and semantic graphical actions to produce biomedical diagrams will reduce the effort required and improve the quality of this visual form of scientific communication.

## Introduction

Pathway diagrams and other molecular “cartoons” are widely used to summarize and communicate biomedical research results and hypotheses. Their nearly universal appearance in molecular biomedical publications clearly indicates the critical role they play in contemporary science. A recent survey of pathway diagrams (a subset of molecular cartoons) identified 64,643 pathway figures published between 1995 and 2019 with 1,112,551 mentions (denotations) of 13,464 unique human genes participating in a wide variety of biological processes (Hanspers et al., 2020). While most of these diagrams are consumed digitally (as PDFs, web pages, etc.), their structure and the tools used to create them remain firmly rooted in the print era. Most such diagrams are produced with non-specialized drawing software such as PowerPoint, and remain closely tied to the model of drawing on paper. Existing software for producing such drawings operate primarily on generic structures (e.g., node/arc diagrams), and some provide domain-specific glyphs or templates (such as those in the Systems Biology Markup Language (SBML) (Hucka et al., 2018) or in commercial tools such as BioRender (BioRender, 2022). Drawing operations are also generic, such as grouping or aligning drawing objects, without specific relevance for biological location or function. The resulting diagrams lack a consistent level of detail and terminology which can make them difficult to interpret across biological topics.

Knowledge graphs (KGs) are structured data models that computationally represent knowledge in the form of interlinked entities—objects, events, situations or abstract concepts, and the relationships between them. All entities and relationships in a knowledge graph are grounded to a formal meaning representation, generally a computational ontology such as the Gene Ontology (Ashburner et al., 2000). The Google knowledge graph (Singhal, 2012) is perhaps the best known and most widely used example. Recently, substantial resources have gone into creating rich, well-structured, and ontologically grounded biomedical knowledge graphs (Callahan et al., 2019). Such KGs include detailed representation of molecular pathways similar to those found in REACTOME (Jassal et al., 2020), disease-specific knowledge integrations such as KG-COVID-19 (Reese et al., 2021), linkages between model organisms, genes and phenotypes including the Monarch Initiative (Shefchek et al., 2020), and broadly crosscutting integration of medically relevant molecular mechanisms such as SPOKE (Nelson et al., 2019), and PheKnowLator (Callahan et al., 2020). These KGs provide broad, detailed, and carefully curated information about molecular biology.

In principle, the rich information available in biomedical KGs could be used to support the creation of molecular cartoons. However, the communicative intent of a molecular cartoon differs significantly from the more detailed and exhaustive design required for computationally effective KGs. Direct visualization of the contents of such knowledge-bases is of modest utility; the information contained is both too detailed and too generic for the communication needs that molecular diagrams serve. Here, we characterize the differences between molecular cartoons and KGs, and present several novel computational approaches for using knowledge graphs to both identify appropriate information within a KG, and transform it into depictions that are effective visualizations. We demonstrate the utility of these techniques through a set of case studies reverse engineering multiple, diverse, peer-reviewed molecular cartoons using KGs. The techniques we describe form the basis of a new paradigm for producing molecular diagrams, making scientific communication faster and easier to produce, and potentially of higher quality.

**FIGURE 1:**
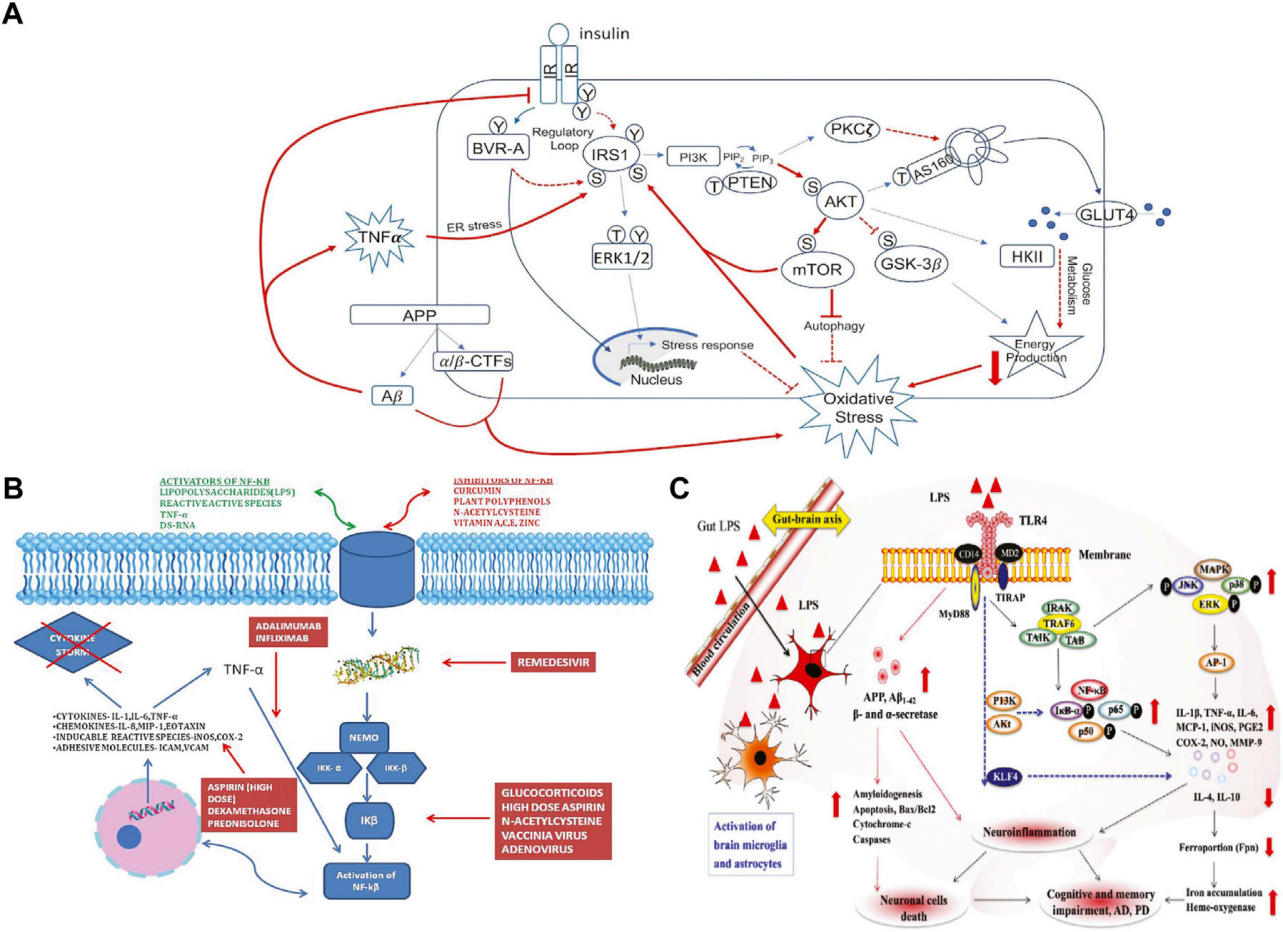
Figures selected from the literature. **(A)** From Figure 2 “Schematic representation of insulin signaling with highlighted in red pathways found to promote brain insulin resistance in AD and DS.” in (Dierssen et al., 2020). **(B)** From Figure 2 “Mechanism of action of various drugs used in COVID-19 and how they inhibit the NF-κB pathway” in (Hariharan et al., 2021). **(C)** From Figure 3 “Gut–brain axis exacerbates neurological disorders through gut-microbiota-derived molecular patterns.” in (Suganya and Koo, 2020).

Our methodology introduces the idea of *semantic graphical actions*, that allow for the complex and abundant representation of information in a knowledge graph to be transformed into a useful diagram over a given domain. These semantic graphical actions search, select, filter, transform, group, arrange, connect, extract, and augment relevant subgraphs from KGs. Our focus is on the semantic content of the figures, not the layout, glyphs, or aesthetic aspects. Our case studies are rooted in three molecular cartoons from published pathway figures pertaining to Down Syndrome, COVID-19, and microbiome-mediated gut-brain connections (Figure 1).

## Methods

**FIGURE 2:**
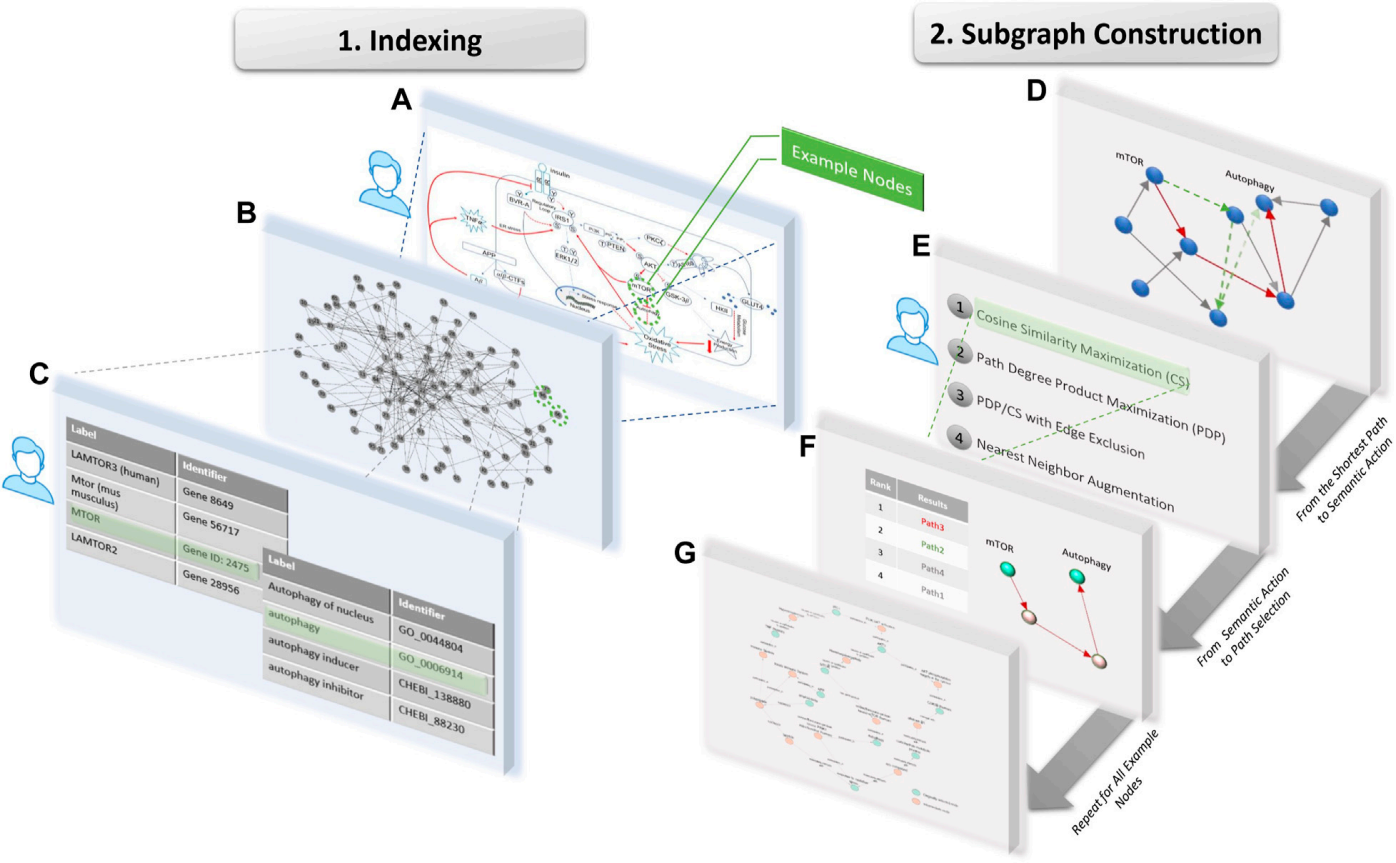
Workflow used to extract nodes from an original example cartoon, where steps requiring user input are indicated with a person icon. During Indexing, **(A)** specific concepts, in this case mTOR and autophagy, are selected from the original cartoon as user input, **(B)** the input concepts are mapped to a sets of nodes in the given KG using partial string matching **(C)** then the user selects 1 node to represent each concept. During Subgraph Construction, **(D)** all shortest paths are identified between the given pair of nodes, and **(E)** the user selects a semantic action which **(F)** ranks the paths and selects the highest ranked path. **(G)** This pipeline is repeated for all example node pairs to produce the resulting subgraph for visualization.

We developed a pipeline to systematically index, search with semantic constraints, and visualize a given molecular pathway using both the PheKnowLator and the KG-COVID19 knowledge graphs (Figure 1). The purpose of this pipeline is to demonstrate the potential of KGs to support molecular cartoon generation by recapitulating the production of several published cartoons. This proof-of-concept pipeline allows for the user to manually select concepts of interest from the original figure, and manually perform the indexing step to map these concepts to KG nodes, such that any cartoon example can be evaluated with this method. Given a manually curated cartoon diagram, represented as a subset of connected concepts as the “source” node and “target” node of interest, the pipeline will allow a user to generate a network visualization using Cytoscape that represents the original concepts and any intermediate nodes connecting these concepts (Shannon et al., 2003). The pipeline, described in Figure 2, begins with an input file that describes the entities to be included (from the original diagram) and some of their connections. Those nodes and target edges are used to index into and explore a KG to demonstrate a process that could have produced the cartoon. The input concepts are mapped to nodes that exist in the KG using fuzzy substring matching. However, many nodes in a KG might match such a string (e.g., genes, variants, gene products and biological processes often share closely related names) so further processing must choose the specific desired KG entity (a semantic action which we term *indexing* into the graph). Next, shortest paths are found between each source and target node pair using a breadth-first search algorithm with unweighted edges; note that there are generally many paths with the same length between a pair of nodes. These paths are then filtered or prioritized by various potential semantic graphical actions to produce a schematic of a final figure.

We describe four semantic graphical actions which can be used to prioritize among paths between a given pair of nodes. The first is maximization of cosine similarity between intermediate nodes and the target (referred to as CS). The score for each path is calculated by summing the cosine similarities between the target node vector embedding and the vector of each other node along the path. The higher this score, the closer all nodes on the path are to the target. Vector embeddings are generated using Node2Vec (Grover and Leskovec, 2016) (with parameters: walk length 10, number of walks 20, window 10, number of dimensions 128). This semantic action biases the search towards paths whose intermediate nodes are all semantically close to the target. Such paths can be thought of as getting semantically close to the target as early in the path as possible, which might prioritize scientifically meaningful results.

The next graphical action is maximization of path-degree product (PDP) (Himmelstein and Baranzini, 2015) (Eq. 1), which prioritizes paths that consist of lower degree intermediate nodes. Although calculated based on the structure of the network rather than its semantics, this heuristic also tends to produce meaningful paths by avoiding hubs (high degree nodes).

$$path\;degree\;product=\prod_{d\epsilon D_{path}}d{=}^{-w}$$ (1)

For example, noting that a node has relation “only_in_taxon” to “Homo sapiens” is unlikely to be interesting, “Homo sapien” is a very higher degree node (connected to many other nodes). This action can therefore draw upon paths that describe molecular interactions as opposed to simple, known facts.

Another semantic action, used in combination with the first two, are explicit requirements or prohibitions of edge classes on a path. As required or excluded edges may eliminate all paths between a pair of nodes in some cases, it can also be implemented as a soft constraint that is ignored when no paths satisfying it can be found. This action is paired with either the CS or PDP ranking algorithm for subgraph construction (referred to as EE).

The final semantic action is to augment this KG with a semantically constrained nearest neighbor search. This search identifies immediate neighbors of extracted nodes and filters to only include those of specified ontological category (e.g., process or drug). The result introduces information contained in the KG beyond the subgraph originally indexed, adding other relevant concepts and relationships. For example, the identification of drugs that affect proteins in a molecular pathway.

The inputs to the pipeline are a file describing the desired nodes and some target connections for the cartoon and the KG (here, two files of node labels and triples using the PheKnowLator or KG-COVID19 format). An interactive indexing interaction helps a user index to the specific intended nodes in the KG (Figure 2, Indexing). Then a subgraph of the KG is generated that includes each node from the input file, and all edges and additional nodes required to connect the inputs based on the selected semantic graphical actions (Figure 2, Subgraph Construction). This subgraph is a semantic schematic of a recapitulated diagram and can be visualized, e.g., in Cytoscape.

To demonstrate the potential of generating cartoon schematics using KGs, we ran the above pipeline on concepts selected from each of the 3 figures depicted in Figure 1, and then compared the resulting subgraphs generated by the various semantic graphical actions used to connect them to the original cartoon. To demonstrate that this tool was agnostic to the KG used, we applied the pipeline to examples using either PheKnowLator or KG-COVID-19 (latest versions as of 9/1/2022) depending on the example content (PheKnowLator for Figure 1A [Insulin Resistance in Down syndrome] and 1c [Neuroinflammation], KG-COVID-19 for 1b [COVID-19 Drugs]). This build of PheKnowLator consisted of 780,753 nodes linked by 5,072,030 edges, with an average degree of 12.99. KG-COVID-19 was more dense with 544,600 nodes linked by 24,145,561 edges and an average degree of 88.67. The goal is a general demonstration of the potential to use KGs to facilitate the production of detailed and accurate molecular cartoons from a simple and incomplete description of desired contents of such a cartoon.

## Results

### Indexing information from knowledge graphs

**FIGURE 3:**
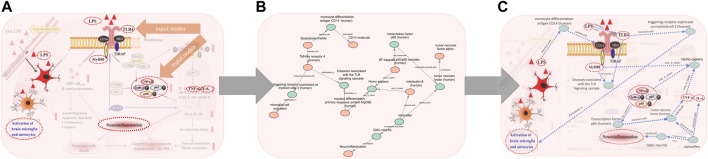
Visualization of the process of generating a subgraph for the Figure 1C example. **(A)** Selection of concepts from the original cartoon to index according to the chosen KG, **(B)** resulting subgraph after semantic actions and path search through the graph to recapitulate the edges between the original nodes, **(C)** the same information from **(B)** though shown with the same artistic portrayal as the original cartoon, with intermediate nodes highlighted.

A semantic system to create cartoons should be able to take simple inputs of key concepts and provide tools to identify relevant concepts in a KG and productively augment those inputs as desired. To demonstrate this potential, we selected as inputs just a few key concepts from each of the target examples shown in Figure 1. Identifying relevant nodes in a KG (“indexing”) requires approximate matching, since the labels in the original figures do not align precisely with the node labels in the KG. The same would likely be true from unconstrained user inputs. The indexing task can be complicated by having many potential matches (e.g., isoforms or variants with similar names and other textual ambiguities), or even by finding no good matches. The result of these figure transformations is portrayed in Figure 3.

For Figure 1A, we selected as inputs only a subset of the nodes in the graph: IRS1, AKT, mTOR, GSK3-beta, autophagy, glucose metabolism, oxidative stress, APP, amyloid beta oligomers, and TNF-alpha. Indexing challenges arise immediately: Searching for TNF-alpha using the string “TNF” in the PheKnowLator KG returns 625 possible matches, including the gene of interest, many known mutations, the transcribed proteins, and several GO terms. Similarly, a search for the string “IRS1” returned 16 possible inputs including the gene and mutations. Sometimes matching is unique, but not string identical. For example, the GO biological process term most closely related to oxidative stress is actually “response to oxidative stress”. In addition to having multiple matches, it is also possible that input labels fail to match any nodes in the graph. For example, there is no generic “AKT” node in PheKnowLator, only the three particular isoforms, AKT1, AKT2, and AKT3.

**FIGURE 4:**
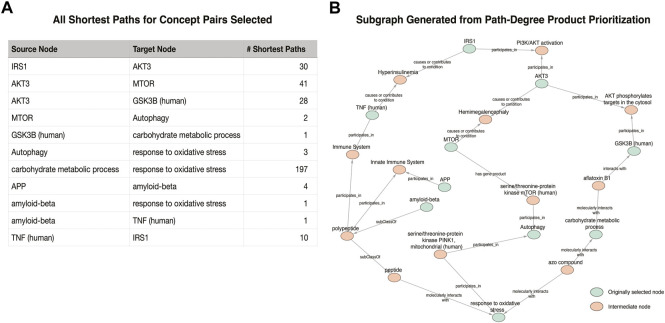
Visualization of the subgraph generated for the Figure 1A example. **(A)** The number of shortest paths found for each pair identified in the original cartoon. **(B)** The result of the Path-Degree Product based ranking algorithm that prioritized one path for each original pair of nodes.

For Figure 1C, the original figure showed how NF-kB signaling results in the production of pro-inflammatory cytokines such as IL-6. Rather than selecting NF-kB as a concept to search in the KG, NF-kappaB p50/-65 complex was selected, as it is the activated form of this signaling process. Indexing variability in identifying a node also can have a significant effect on the paths identified in semantic search. Automatic identification of the best among many related nodes for cartooning is an open question; we used manual selection, and some experimentation to find the best results. One issue is constraining the number of tied paths found in search. The number of paths found *via* shortest path search varied from 1 up to 197 (between “carbohydrate metabolic process” and “response to oxidative stress” in example Figure 1A). Figure 4 illustrates the kinds of paths (and their ambiguities) found between the target nodes in the Figure 1A example, and a visualization of the extracted KG subgraph that forms a figure schematic.

**FIGURE 5:**
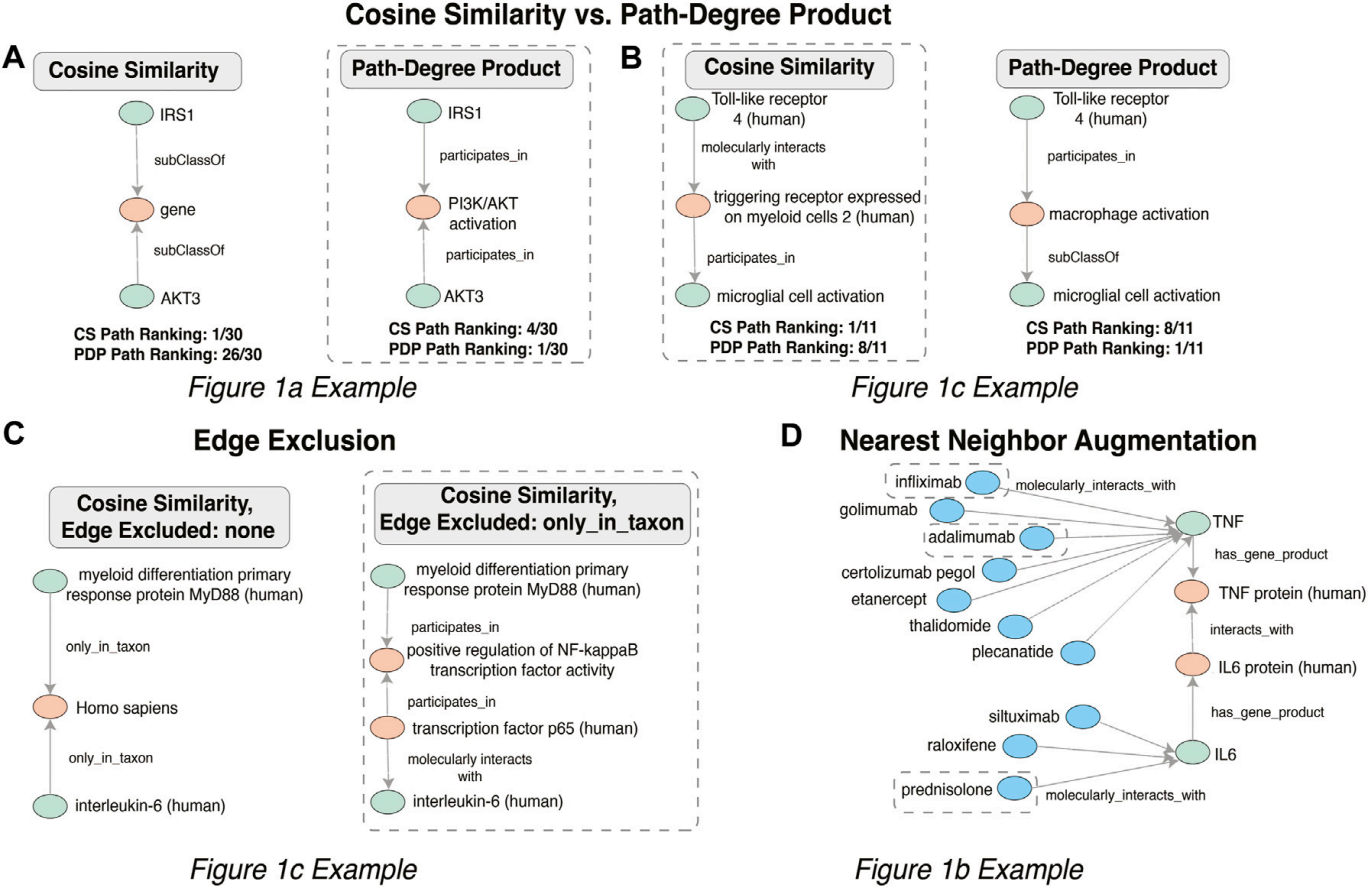
Changes in intermediate nodes between given source and target concepts when the Cosine Similarity **(A)** and Path-Degree Product path ranking algorithm **(B)** were applied, when Edge Exclusion **(C)** was applied and when nearest neighbor augmentation **(D)** of drugs was applied, with those relationships that aligned with the original example Figure 1B circled.

**FIGURE 6:**
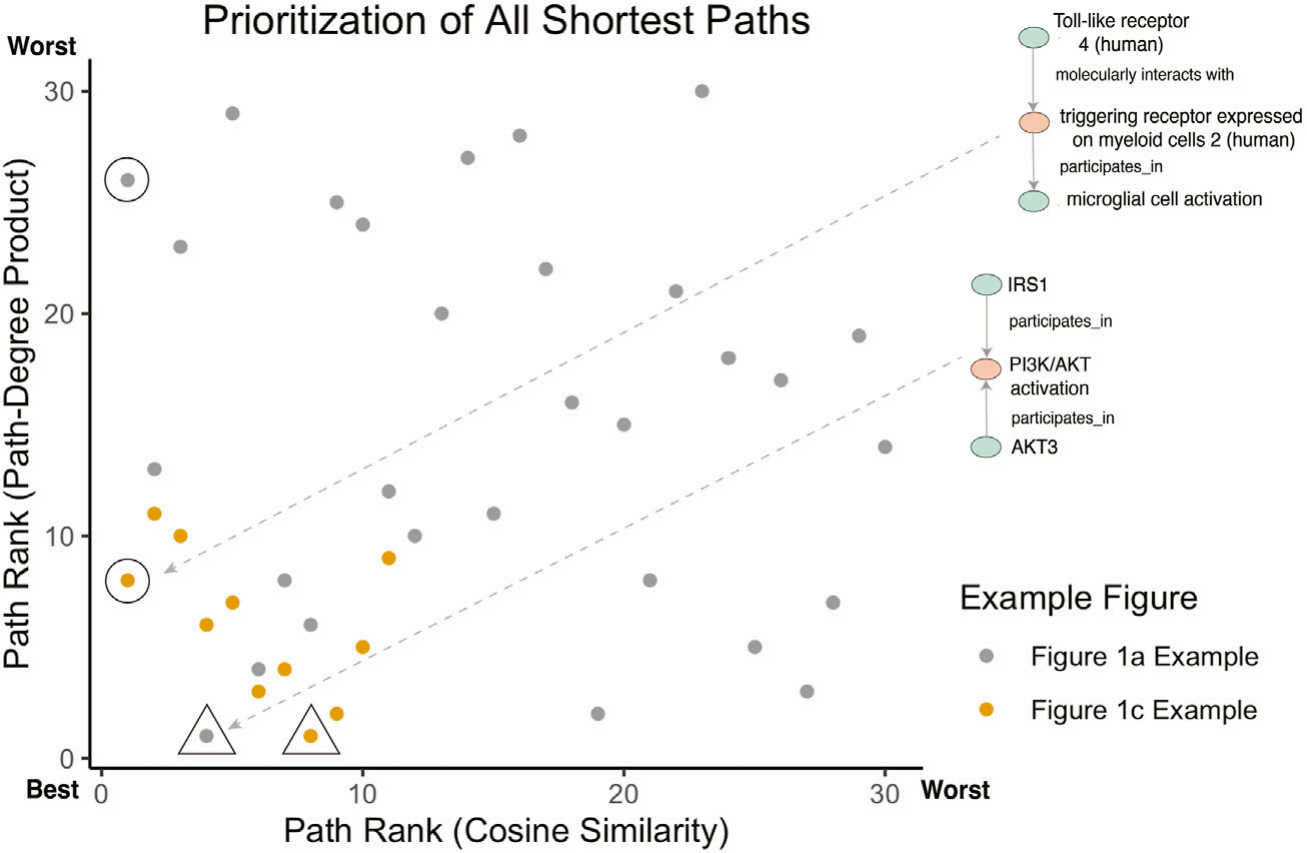
Comparison of path prioritization algorithms. For the Figure 1A example, the rank of all paths between IRS1 and AKT3 is shown (as depicted in Figure 4A). For the Figure 1C example, the rank of all paths between Toll-like receptor 4 (human) and microglial cell activation is shown (as depicted in Figure 4B). Annotated points identify the highest ranked path for Cosine Similarity (circles) and Path-Degree Product (triangles) to highlight the differences in ranking. The Figure 1B example is not included as all pairs had only between 1 and 2 shortest paths.

Figure 4 illustrates the meaningful differences among the various semantic actions for prioritizing paths. The path-degree product prioritization effectively selected intermediate nodes that tended to have a more insightful connection between the source and target nodes. In the Figure 1A example, the intermediate node changes from “gene”, a general high degree node, to “PI3K/AKT activation” which is more aligned to the concepts being portrayed in the original cartoon (Figure 5A). In another example, the cosine similarity ranking selected the more relevant node “triggering receptor expressed on myeloid cells 2 (human)” over “macrophage activation” selected by path-degree product (Figure 5B). The cosine similarity and path-degree product ranking algorithms generally rank paths in uncorrelated order of one another, supporting the idea that each algorithm may serve different use cases (Figure 6).

Semantic criteria to include or prohibit specific nodes and edges had a major effect, sometimes eliminating mundane, uninspiring paths in favor of potentially more mechanistically interesting ones. In the Figure 1C example, the path between “myeloid differentiation primary response protein MyD88 (human)” and “interleukin-6 (human)” avoided “only_in_taxon” by instead going through NF-kappaB transcription factor activity, which is a known precursor to IL-6 production (Figure 5C). The specification of particular semantic types required or prohibited along a path clearly enabled selection of a more relevant and interesting path in this case.

Nearest neighbor augmentation was applied to the Figure 1B example. The goal was to identify drugs interacting with molecules in subgraph, as included in the original figure. Therefore, the neighbors were constrained to only include nodes associated with the ontological category ‘drug’ (taken from the DrugCentral (Avram et al., 2021) and Pharmacogenomics Knowledgebase (Thorn et al., 2013). This added 113 drug nodes and 148 edges to the subgraph. The added drug nodes include 4 of the treatments highlighted in Figure 1B. Specifically, infliximab and adalimumab were identified as interacting with TNF, prednisolone with IL-6 (Figure 5D), and dexamethasone with the intermediate node PTGS2.

## Discussion

This set of case studies shows that KGs can be used to take simple and incomplete descriptions of the desired contents of a molecular cartoon, and transform them into accurate, consistent and detailed schematics. Here, we define accuracy as the recapitulation of input nodes. That is, the input and output contain the same nodes, found *via* the indexing step, and we identify at least one path between them (Figure 4). Consistency refers to the reproducibility of semantic relations between concepts. Using our method of automated graph extraction ensures that researchers are using a standardized system for node and edge semantics (i.e., provided they start from the same KG). A researcher may then collapse or expand nodes and pathways to reach the desired level of abstraction for communicating their claims.

In terms of detail, we started with the information summarized in the cartoon and expanded the subgraph based on relations in the knowledge graph. In each case, our approach included intermediate nodes beyond those in the initial search (Figure 3 and Figure 4). For example, Figure 1A was recapitulated with the intermediate node mitochondrial serine/threonine-protein kinase PINK1 between autophagy and oxidative stress. PINK1 is a protein that contributes to autophagy (Chu 2010; Bordi et al., 2019). Additional detail was also added to Figure 1C. The output subgraph included the specificity of monocyte differentiation antigen CD14 in humans as the receptor for LPS, as opposed to the overarching CD14 molecule class. This subgraph also provides more context for the proinflammatory process by including the intermediate triggering receptor expressed on myeloid cells 2 (TREM-2), a neuroprotective protein involved in the activation of microglia that results from TLR4 activation (Rosciszewski et al., 2018; Hu et al., 2021). Thus, the extracted subgraphs for each figure included more detailed explanations of the mechanistic interaction between the input nodes.

While our cartoon reverse engineering pipeline is not intended as a widely used tool, we intend the proof-of-concept to demonstrate the potential for KGs and graphical semantic actions to underpin a new generation of semantically aware molecular cartooning technology. For instance, a researcher with a list of genes differentially expressed in a disease area may wish to explain this in the context of other biological entities. Future software related to this pipeline could be used to add innovative functionality not currently supported by existing enrichment and/or drawing tools.

**FIGURE 7:**
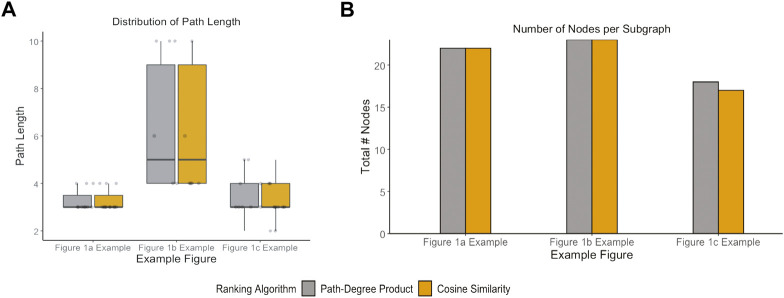
Network properties of subgraphs generated by each path ranking algorithm. **(A)** Path length for all pairs existing in each example figure **(B)** and total number of nodes that exist in the subgraph for each example figure.

**FIGURE 8:**
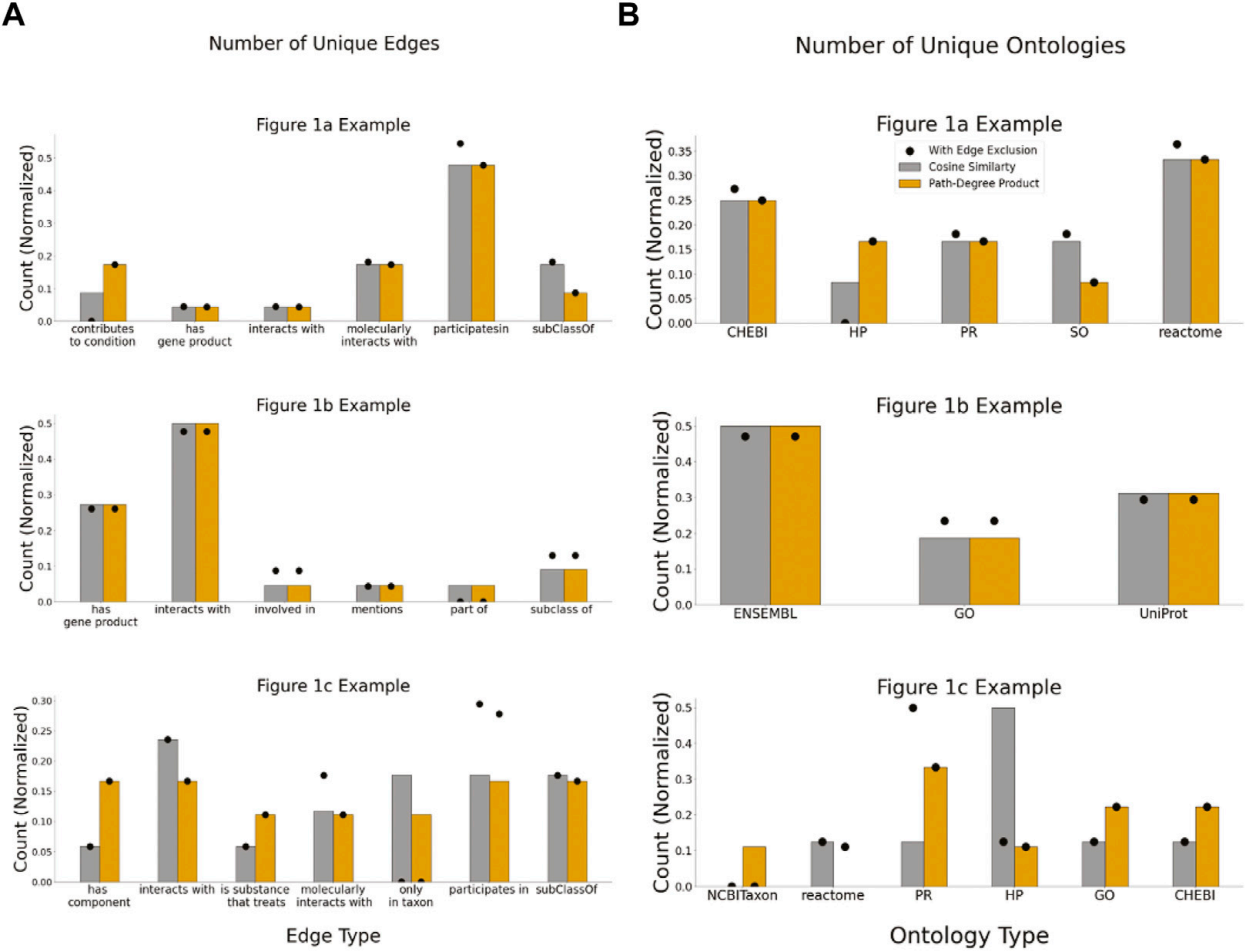
Semantic properties of subgraphs generated by each path ranking algorithm. **(A)** The number of unique edge types within each subgraph generated **(B)** the number of unique ontologies that make up each subgraph generated.

These case studies allowed for quantitative assessments of two applied KGs that may be indicative of more general issues. For example, the two different KGs had distinct topologies: the average path length for the Figure 1B example extracted from KG-COVID19 was longer (Figure 6A) than the average path lengths in the examples that used PheKnowLator. While the number of nodes per subgraph was not significantly different between the cosine similarity and the path-degree product prioritization algorithm (Figure 7B), it is clear that the semantic content of each of the graphs varied, particularly in the Figures 1A,C examples. The edge “has component” has more instances in the subgraph generated with path-degree product prioritization, while “interacts with” and “only_in_taxon” has more instances in the subgraph ranked by cosine similarity (Figure 8). This is likely due to higher degree nodes being more prominent in the latter two edge types. Additionally, intermediate nodes in the Human Phenotype (HP) ontology were more abundant in the cosine similarity subgraph, while nodes in the Protein ontology (PR) were more abundant in the path-degree product subgraph. As we studied only three cases, it would be premature to draw conclusions about the engineering of general tools, but it is clear that there are interesting phenomena related to semantic visualization yet to be explored. In the future, expert review of the resulting subgraphs could serve as a qualitative evaluation of this method for KG-based figure generation.

This work also illustrated some open challenges in generating molecular cartoons from KGs. For example, how to manage directionality in KGs and cartoons. Cartoons often indicate directionality and positive or negative influences *via* arrows and various arrowheads. Our searches were done without considering direction, which increased the number of potential pathways to be discriminated among, and includes paths where the directionality does not connect the source and target (e.g., both source and target are directionally linked to a third node). Another challenge is integrating new findings into the resulting cartoons. For example, identifying the nearest neighbors of drug ontologies in the COVID-19 subgraphs added 113 potential treatments of which only 4 were included in the published figure. Those 4, along with the others in the published figure, have shown efficacy in clinical trials and this information has not yet been included in KG-COVID19.

An open challenge in automated molecular cartoon generation is the integration of existing resources and databases. For example, one of the difficulties in *indexing* was the differential mappings for multiprotein families like AKT between the cartoon and KG. Using standardized entity linking resources [e.g., FamPlex (Bachman et al., 2018) for multi-protein families] as opposed to user selection will improve consistency in extracted subgraphs. Moreover, there are multiple existing pathway databases built from decades of expert curation of molecular interactions that serve as a gold standard for pathway representation [e.g., Reactome, WikiPathways (Martens et al., 2021) and Pathway Commons (Cerami et al., 2011)]. In this work, we suggest broadly applicable semantic actions that arrive at a detailed molecular subgraph. A future direction of evaluating KG-based results against existing pathway databases would provide a useful benchmark for the proposed methodology. For example, we could compare the output graphs extracted using CS, PDP, or any future semantic actions against a set of curated pathways containing the input nodes.

Not all open challenges indicated by this work are semantic. The performance of the algorithms may be improved by filtering the neighbor list during the shortest path search, rather than generating all shortest paths and later prioritizing paths. The current visualization uses Cytoscape, which is a software that creates graphical representations of a network object consisting of nodes and edges. There is an extensive ecosystem of plugins in Cytoscape to curate an affordance for scientific communication. Our approach builds upon and adds to this ecosystem through the application of the semantic schema in KGs to the creation of figures. Future versions of the method will employ glyphs to facilitate the communication of biological concepts.

These case studies demonstrate the potential of KGs and semantic graphical actions to provide innovative new functionality for molecular cartooning. Knowledge-based and semantic tools for cartoon construction could reduce the difficulty of creating high quality scientific communication, and are ripe for future development.

## Data Availability

The repository used for analysis can be found in our git-hub repository [https://github.com/UCDenver-ccp/Cartoomics]. The knowledge graphs used for this study can be found in the PheKnowLator Google Cloud Storage Bucket or the kg-covid19 Knowledge Graph Hub, which are listed in the ReadMe.
